# AGED MICE ARE SUSCEPTIBLE TO CARDIAC HYPERTROPHY AFTER 1 WEEK OF CONSUMING A HIGH SUGAR DIET

**DOI:** 10.1093/geroni/igz038.402

**Published:** 2019-11-08

**Authors:** Ana P Valencia, Jeremy Whitson, Rudolph Stuppard, Gustavo Valencia, Peter Rabinovitch, David Marcinek

**Affiliations:** University of Washington, Seattle, Washington, United States

## Abstract

Over 80% of American adults exceed their daily recommended intake of sugar (<10% kcal). While habitual sugar consumption is associated with an increased risk for diabetes and cardiovascular disease, less is known about the effects of short-term sugar consumption on metabolic health, particularly in the elderly. The purpose of this study was to test whether aged hearts are more susceptible to pathology following a short-term high sucrose (HS) diet. Specific goals were to: A) determine the effects of a 1-week HS diet exposure on the hearts of 5 month-old and 24 month-old mice; and B) test if the mitochondrial targeted peptide SS-31 can protect against HS-diet induced effects. Male CB6F1 mice were placed either on standard chow or HS diet after 1 week of receiving saline (control) or SS-31 through osmotic pumps. Heart function was assessed in vivo through echocardiography before and after treatments. One week of HS induced significant cardiac hypertrophy in the old mice compared to age-matched chow controls. Treatment with SS-31 prevented this HS induced hypertrophy. Young hearts were smaller than in the old, but size was unaffected by diet or SS-31. We observed no effect of HS (with or without SS-31) on respiration or H2O2 production in isolated mitochondria from hearts using high-resolution respirometry. These data indicate that only 1-week exposure to HS diet is enough to exacerbate cardiac hypertrophy in aging mice, but factors other than heart mitochondrial ROS may mediate this effect.

